# Supplementary material for: Human scent signature on cartridge case survives gun being fired: A preliminary study on a potential of scent residues as an identification tool
Source: PLoS One. 2023 Mar 22;18(3):e0283259. doi: 10.1371/journal.pone.0283259 (PMC10032514; doi:10.1371/journal.pone.0283259)
Supplement: S1 File — (PDF) [file pone.0283259.s001.pdf]

S1 Calculations

All user-written scripts used through this article is published at:

https://github.com/nickvusko/2D_data_chrom_automation.git

https://github.com/nickvusko/Machine_learning_scent.git

S1.1 Principal Component Analysis

The PCA model was built on PCA class imported from Scikit-learn module using single value decomposition algorithm. The n_compopnents parameter was set to 7, and svd_solver was set to full. The data were scaled using StandardScaler imported from the very same module.

S1.1.1 PCA – Model-Based Analysis of Fired Cartridge Samples

Explained variance ratios for 7 principal components:

PC1: 0.32026149, PC2: 0.27694163, PC3: 0.18796439 PC4: 0.05812328, PC5: 0.05232891, PC6: 0.0336314, PC7: 0.03008165

Explained variance for 7 principal components:

PC1: 22.70945118, PC2: 19.63767955, PC3: 13.32838435, PC4: 4.12146928, PC5: 3.71059541, PC6: 2.384772, PC7: 2.13306268

Singular values for 7 principal components:

PC1: 15.80518785, PC2: 14.6974309, PC3: 12.10835364, PC4: 6.73321335, PC5: 6.3887831, PC 6: 5.1217665, PC7: 4.84393326]

Noise variance for the model:

0.5767352917735807

S1.1.2 PCA – Model-based analysis of standard samples

Explained variance ratios for 7 principal components:

PC1: 0.22390826, PC2: 0.19713889, PC3: 0.12702732, PC4: 0.09033323, PC5: 0.0812571, PC6: 0.05085097, PC7: 0.03428233

Explained variance for 7 principal components:

PC1: 13.04022249, PC2: 11.4811975, PC3: 7.39796071, PC4: 5.26092863, PC5: 4.73234285, PC6: 2.96151627, PC7: 1.99657327

Singular values for 7 principal components:

PC1: 24.49184016, PC2: 22.98118981, PC3: 18.44738986, PC4: 15.55643652, PC5: 14.75424587, PC6: 11.67175001, PC7: 9.58344252]

Noise variance for the model:

0.28420971787062344

S1.2 Classification models

S1.2.1 KNN – fired cartridges

Best parameters: KNeighborsClassifier(n_neighbors=1, weights=uniform)

Table S1.2.1 Classification report of KNN applied on cartridge samples.

	Precision	Recall	F1-score	Support
Vol1	1.00	1.00	1.00	2
Vol2	1.00	1.00	1.00	1
Vol4	1.00	1.00	1.00	1
Weighted avg	1.00	1.00	1.00	4

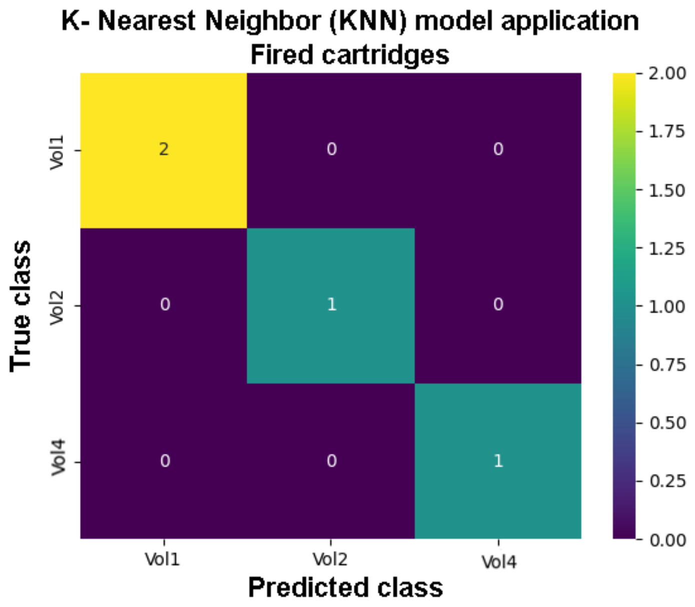

Fig S1.2.1. Confusion matrix of KNN applied to cartridge samples.

S1.2.2 KNN – scent samples

Best parameters: KNeighborsClassifier(n_neighbors=4, weights=uniform)

Table S1.2.2 Classification report of KNN applied on scent samples.

	Precision	Recall	F1-score	Support
Vol1	0.60	1.00	0.75	3
Vol2	1.00	0.75	0.86	4
Vol3	1.00	1.00	1.00	1
Vol4	1.00	0.50	0.67	2
Vol5	1.00	1.00	1.00	3
Weighted avg	0.5	0.5	0.85	13

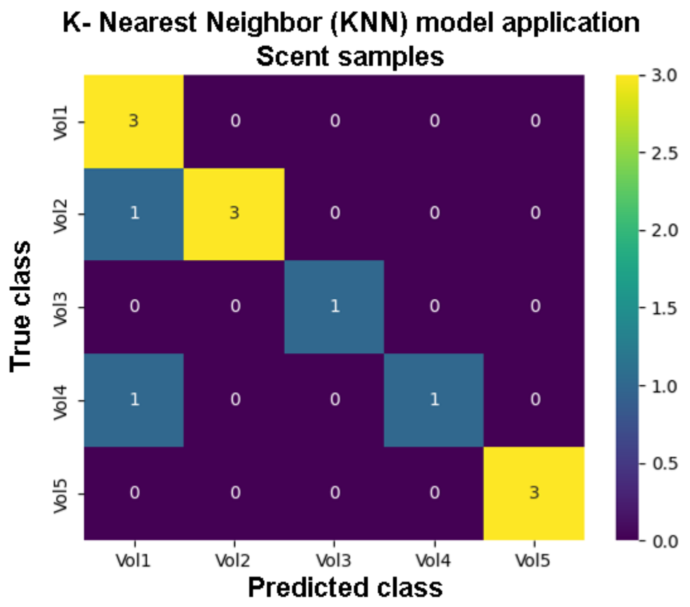

Fig S1.2.2. Confusion matrix of KNN applied on scent samples.

S1.2.3 KNN – scent sample (PC1, PC2 and PC3 as an input)

Best parameters: KNeighborsClassifier(n_neighbors=4, weights=uniform)

Table S1.2.3 Classification report of KNN applied on first 3 component of PCA dimensional reduction of scent samples variables.

	Precision	Recall	F1-score	Support
Vol1	0.75	1.00	0.86	3
Vol2	1.00	1.00	1.00	4
Vol3	1.00	1.00	1.00	1
Vol4	1.00	0.50	0.67	2
Vol5	1.00	1.00	1.00	3
Weighted avg	0.94	0.92	0.92	13

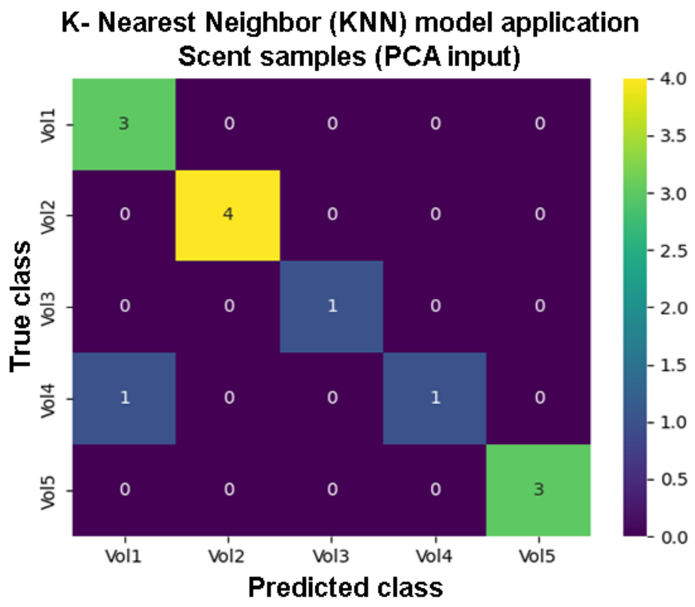

Fig S1.2.3. Confusion matrix of KNN applied on first 3 component of PCA dimensional reduction of scent samples variables.

S1.2.4 RNN – fired cartridges

Best parameters: RadiusNeighborsClassifier(radius=31, weights='distance')

Table S1.2.4 Classification report of RNN applied on cartridge samples.

	Precision	Recall	F1-score	Support
Vol1	0.00	0.00	0.00	2
Vol2	1.00	1.00	1.00	1
Vol3	0.00	0.00	0.00	0
Vol4	1.00	1.00	0.50	1
Weighted avg	0.50	0.50	0.50	4

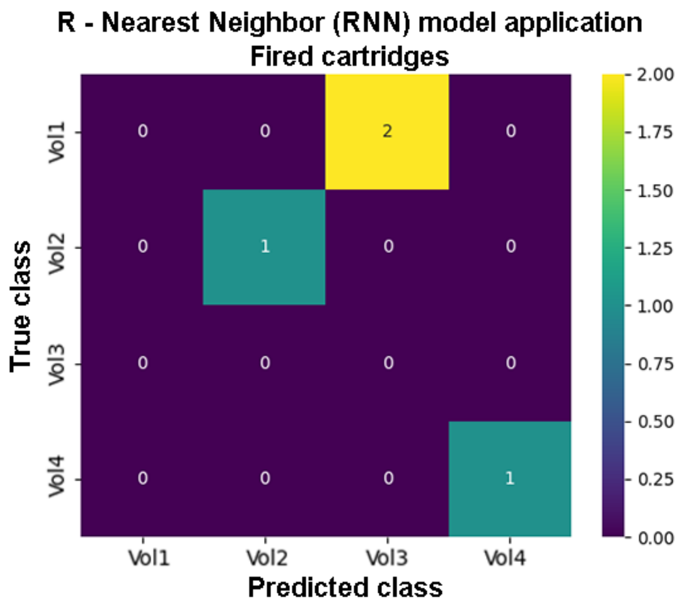

Fig S1.2.4. Confusion matrix of RNN applied on cartridge samples.

S1.2.5 RNN – scent samples

Best parameters: RadiusNeighborsClassifier(radius=21, weights='distance')

Table S1.2.5 Classification report of applied on scent samples.

	Precision	Recall	F1-score	Support
Vol1	0.60	1.00	0.75	3
Vol2	0.00	0.00	0.00	4
Vol3	0.33	1.00	0.50	1
Vol4	1.00	1.00	1.00	2
Vol5	1.00	1.00	1.00	3
Weighted avg	0.55	0.69	0.60	13

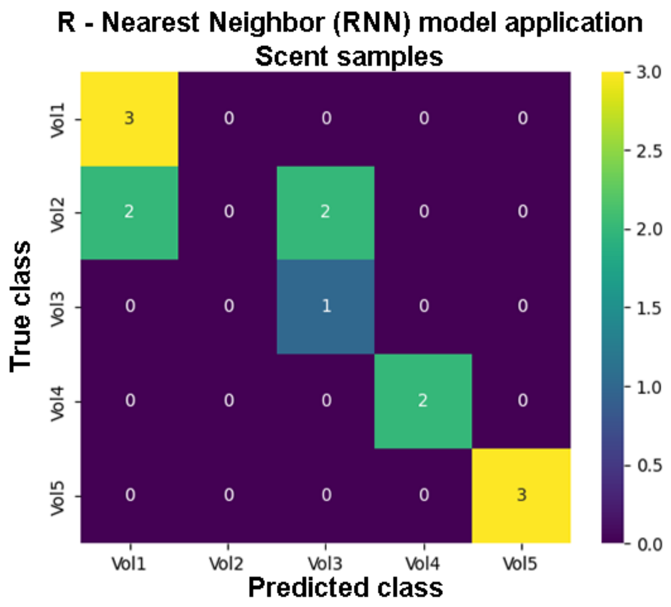

Fig S1.2.5. Confusion matrix of RNN applied on scent samples.

S1.2.6 RNN – scent sample (PC1, PC2 and PC3 as an input)

Best parameters: RadiusNeighborsClassifier(radius=11, weights='distance')

Table S1.2.6 Classification report of RNN applied on first 3 component of PCA dimensional reduction of scent samples variables.

	Precision	Recall	F1-score	Support
Vol1	0.60	1.00	0.75	3
Vol2	1.00	0.75	0.86	4
Vol3	1.00	1.00	1.00	1
Vol4	1.00	0.50	0.67	2
Vol5	1.00	1.00	1.00	3
Weighted avg	0.91	0.85	0.85	13

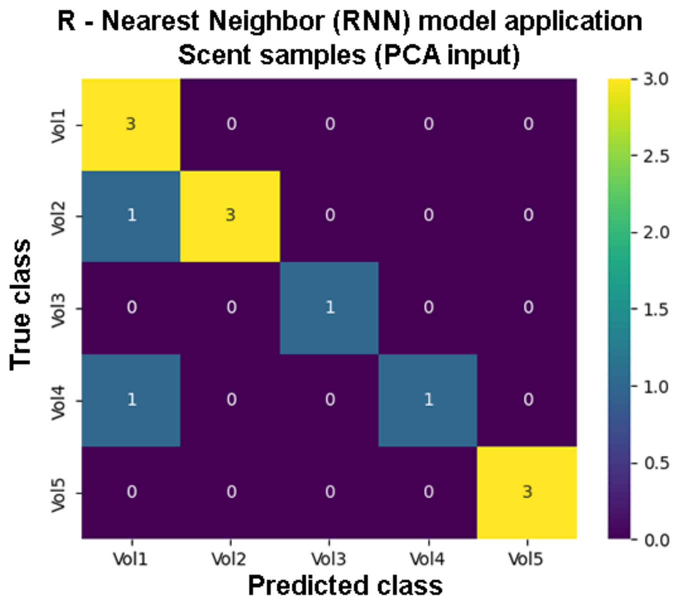

Fig S1.2.6. Confusion matrix of RNN applied on first 3 component of PCA dimensional reduction of scent samples variables.

S1.2.7 RF – fired cartridges

best parameters: RandomForestClassifier(n_estimators=15, max_features=1, bootstrap=True, oob_score=True)

Table S1.2.7 Classification report of KNN applied on cartridge samples.

	Precision	Recall	F1-score	Support
Vol1	1.00	1.00	1.00	2
Vol2	1.00	1.00	1.00	1
Vol4	1.00	1.00	1.00	1
Weighted avg	1.00	1.00	1.00	4

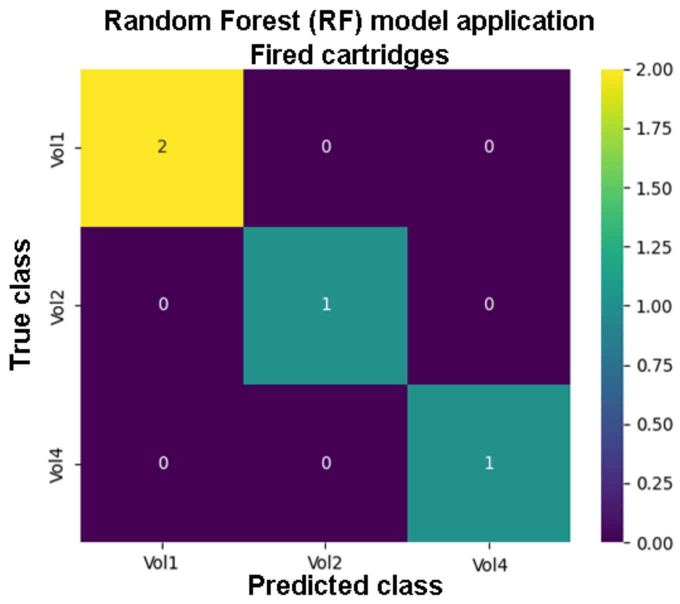

Fig S1.2.7. Confusion matrix of RF applied on cartridge samples.

S1.2.8 RF – scent samples

best parameters: RandomForestClassifier(n_estimators=25, max_features=1, bootstrap=True, oob_score=False)

Table S1.2.8 Classification report of applied on scent samples.

	Precision	Recall	F1-score	Support
Vol1	1.00	1.00	1.00	3
Vol2	1.00	1.00	1.00	4
Vol3	1.00	1.00	1.00	1
Vol4	1.00	1.00	1.00	2
Vol5	1.00	1.00	1.00	3
Weighted avg	1.00	1.00	1.00	13

Confusion matrix: please refer to Fig 6

S1.2.9 RF – scent samples (PC1, PC2 and PC3 as an input)

Best parameters: RandomForestClassifier(n_estimators=105, max_features=1, bootstrap=True, oob_score=False)

Table S1.2.9 Classification report of RNN applied on first 3 component of PCA dimensional reduction of scent samples variables.

	Precision	Recall	F1-score	Support
Vol1	1.00	1.00	1.00	3
Vol2	1.00	1.00	1.00	4
Vol3	1.00	1.00	1.00	1
Vol4	1.00	1.00	1.00	2
Vol5	1.00	1.00	1.00	3
Weighted avg	1.00	1.00	1.00	13

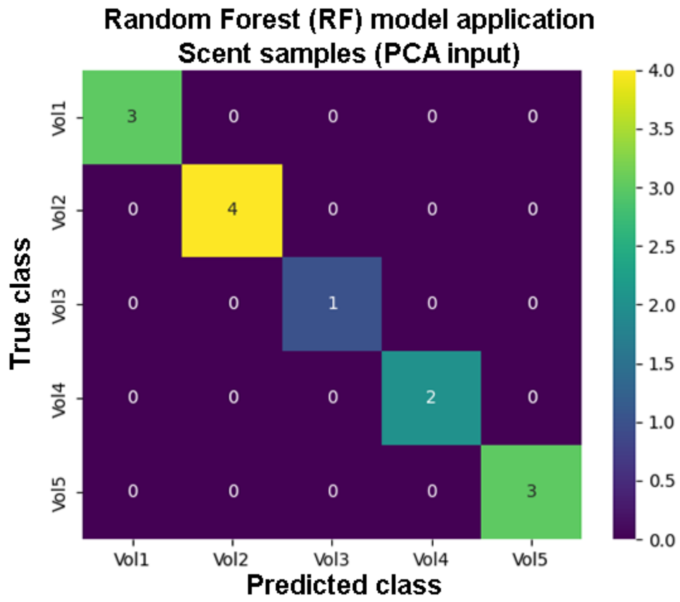

Fig S1.2.9. Confusion matrix of RNN applied on first 3 component of PCA dimensional reduction of scent samples variables.

S1.3 *Spearman's rank correlation*

Python function spearmanr from scipy.stat module was used for the calculation. Please refer to: https://docs.scipy.org/doc/scipy/reference/generated/scipy.stats.spearmanr.html (Access date: 28.02.2022)

S1.4 Cosine similarity

The Python function distance.cosine from the scipy.spatial module was used for the calculation. Please refer to: https://docs.scipy.org/doc/scipy/reference/generated/scipy.spatial.distance.cosine.html (Access date: 28.02.2022)

S1.5 Pearson's correlation

Python script used for calculating the Pearson correlation:
data_array stands for the values of examined sample variables
values_array stand for values of „standard" sample variables
data_mean = data_array.mean()
values_mean = values_array.mean()
data_less = data_array - data_mean

values_less = values_array - values_mean
*numerator = (np.sum(data_less*values_less))*
*denominator = np.sqrt((np.sum(data_less**2))*(np.sum(values_less**2)))*
similarity = numerator/denominator

S1.6 Kendall's tau

The Python function kendalltau from the scipy.stats module was used for the calculation. Please refer to: https://docs.scipy.org/doc/scipy/reference/generated/scipy.stats.kendalltau.html (Access date: 28.02.2022)

S1.7 Test for the significance of the difference between top two Spearman correlation scores

Original code:

```
import numpy as np

import scipy.stats


_SPEARMAN_TOP_2 = [(0.5256, 0.4402), (0.6800, 0.3391), (0.6249, 0.0972), (0.3922, 0.3798), (0.6696, 0.2735), (0.7652, 0.6280), (0.9302, 0.8687), (0.9221, 0.7919), (0.6128, 0.5063), (0.6427, 0.5999)]

n1 = 60

n2 = 60


for comp in _SPEARMAN_TOP_2:

    z1 = 0.5*(np.log((1+comp[0])/(1-comp[0])))

    z2 = 0.5*(np.log((1+comp[1])/(1-comp[1])))

    z_eval = (z1-z2)/np.sqrt((1/(n1-3))+1/(n2-3))

    print(z_eval, scipy.stats.norm.sf(abs(z_eval))*2)
```

where n1 and n2 is the number of peak area ratios, z1 and z2 are the Fisher's transformation of each pair of correlations, *z_eval* is the final *z-score*. The function prints the *z-score* and *p-value* (scipy.stats.norm.sf(abs(z_eval))*2).
