# Supplementary material for: Human scent signature on cartridge case survives gun being fired: A preliminary study on a potential of scent residues as an identification tool
Source: PLoS One. 2023 Mar 22;18(3):e0283259. doi: 10.1371/journal.pone.0283259 (PMC10032514; doi:10.1371/journal.pone.0283259)
Supplement: S2 File — (PDF) [file pone.0283259.s002.pdf]

UNIVERSITY OF CHEMISTRY AND TECHNOLOGY PRAGUE

Consent and instruction of the research participant

Instruction of the research participant:

Dear Sir/Madam,

In accordance with the principles of ethical research[1], I ask you for your consent to your participation in a research project dealing with the possibilities of individual and species identification of persons from the molecular profile of human scent.

Project name: Chemical analysis of human scent for forensic purposes
Project solver: Petra Pojmanová, Ph.D. (petra.pojmanova@vscht.cz), Nikola Ladislavová, MSc. (ladislan@vscht.cz)
Name of the workplace: Department of Analytical Chemistry, Faculty of Chemical Engineering University of Chemistry and Technology Prague
Project manager: prof. Štěpán Urban, Ph.D. (urban@vscht.cz)
Research target: The aim of the research is to find the relationship between the molecular composition of human scent and a given individual or group of people.

Research description: Within this research, a database of skin scent samples from different persons is created, with each person (participant) providing the scent sample repeatedly (at least 10 samples, the time interval between samples is ideally one week). Repeated sampling is carried out in order to capture various fluctuations in the odor profile of the volunteer and so that not only his "average" scent can be sought, enabling individual identification, but also trends can be sought to identify species (sex, age, ethnicity, drug abuse, etc.).

For the purpose of creating a correlation database, each sample also includes the completion of a questionnaire on various aspects that are expected to affect human scent (e.g., sex, age, weight, medications used, drugs, physical or stress load, etc.). It is not obligatory to answer all the questions listed in the questionnaire, if you do not want to answer a question or do not know the answer, then skip the given question. Each questionnaire will be marked with the code assigned to you, while your individual samples will be marked with the code and order of the sample taken (e.g., M1_01, F3_09, etc.). The questionnaire is anonymized (it does not contain information that would allow you to find out your identity). Only the main researchers of this research bound by confidentiality will have access to a separate file containing the names and

[1] The Universal Declaration of Human Rights, Regulation (EU) No. 2016/679 of the European Parliament and of the Council on the protection of individuals with regard to the processing of personal data and on the free movement of such data and repealing Directive 95/46 / EC (General Data Protection Regulation) and other generally binding legal regulations *(such as the Helsinki Declaration adopted by the 18th World Health Assembly in 1964, as amended (Fortaleza, Brazil, 2013), Act No. 372/2011 Coll., on health services and conditions for their provision (Act on Health Services), as amended, in particular the provisions of Section 28 (1) thereof, and the Convention for the Protection of Human Rights and Dignity of the Human Being with regard to the Application of Biology and Medicine: Convention on Human Rights and Biomedicine published under No. 96 / 2001 Coll., If applicable).*

codes of individual volunteers and contacts (telephone or e-mail) to them. This special list will be deleted when the research is completed.

The time required for each sampling is then around 30 minutes. The collection consists of washing hands with uncolored and unscented soap for food purposes, drying hands freely in the air so as not to contaminate them (approx. 5 min), rubbing hands on each other to increase scent production (5 min), and collection (10 min). The collection is carried out by rubbing the sorption material in the palms. The sorption material is most often glass in the form of beads, but another suitable sorbent can be used. The last 10 minutes are then devoted to completing the questionnaire, which consists of 30 questions, of which the first 20 questions are answered only at the first sampling, the remaining 10 questions are always answered. You can look at the template of this questionnaire to get acquainted with the research. There is no known safety risk in this research that would harm the health of the subjects.

After sampling and labeling, odor samples are stored in a refrigerator in a lockable room until analysis. The samples are then analyzed using gas chromatography methods (GC-MS, GC×GC-MS, or GC-FID) or liquid chromatography (LC-MS). Samples can be used to optimize measurement methods. After the measurement, the scent samples are discarded.

The evaluated scent samples will be used together with anonymized data from completed questionnaires to create a correlation database, in which it will be possible to search for relationships between the molecular profile of human scent and a given individual or group of people using various multidimensional methods. The results can be published in professional journals or be part of the final theses while ensuring the anonymity and unidentifiable nature of individual persons. Only anonymized files will be provided to third parties if necessary, but always only for research purposes or grant matters.

The signed "Declaration and informed consent" forms without a code mark will be archived for a period of 5 years.

Participation in this research is voluntary and it is possible to leave the research at any time without giving a reason.

……………………………………………………
Date and signature of the project solver

UNIVERSITY OF CHEMISTRY AND TECHNOLOGY PRAGUE

Statement and informed consent of participants with their involvement in research: Chemical analysis of human scent for forensic purposes

I declare and confirm by my handwritten signature below that I voluntarily agree to participate on the project mentioned above and with the publication of the data obtained. I have had enough time and the opportunity to thoroughly consider all relevant research information, to ask for all relevant information related with participation in research and I have received clear and comprehensible answers to my questions. I have been informed of the right to refuse to participate in the research project or to withdraw my consent at any time without any repression.

Name and surname of the participant: ……………………… Date of Birth: …………..

The place, date: …………………………………………

Signature of the participant …………………………………………

(Indicate if the research participant is under 18 years of age.)

Name and surname of legal representative: …………….…….. Date of Birth: …………..

Relationship of the legal representative to the participant:

The place, date: …………………………………………

Signature of legal representative ……………………………………

Department of Analytical Chemistry, Faculty of Chemical Engineering
University of Chemistry and Technology Prague
Technická 5, 166 28 Praha
Czech Republic, https://uanlch.vscht.cz/?jazyk=en/
