# Supplementary figures and images for: Human scent signature on cartridge case survives gun being fired: A preliminary study on a potential of scent residues as an identification tool
Source: PLoS One. 2023 Mar 22;18(3):e0283259. doi: 10.1371/journal.pone.0283259 (PMC10032514; doi:10.1371/journal.pone.0283259)

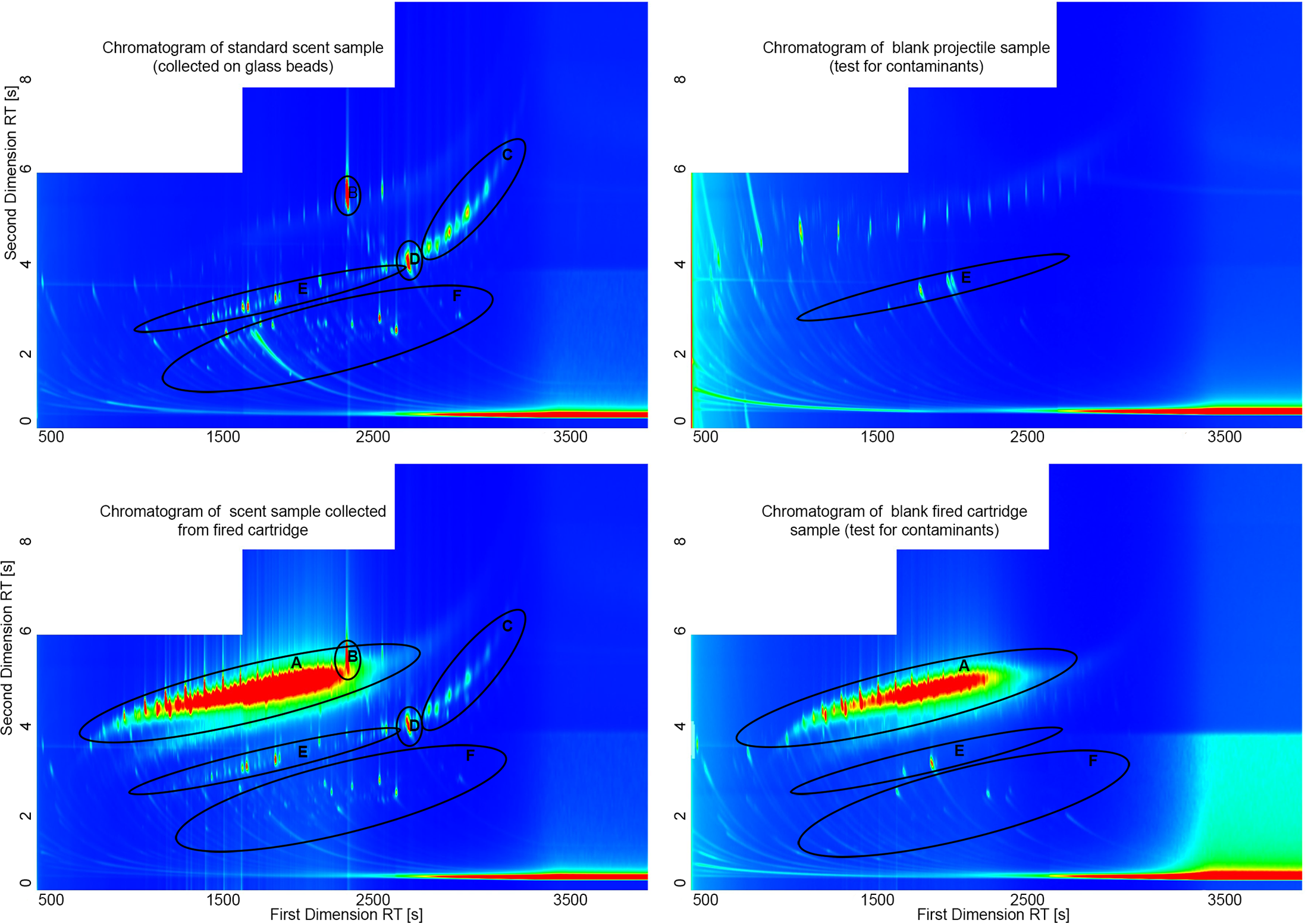

Supplement: S1 Fig — A = area of saturated and unsaturated hydrocarbons (the cluster most likely originated from gun lubricants); B = Squalen; C = Esters of higher fatty acids; D = Cholesterol; E = Ethyl esters of carboxylic acids; F = Heterocycles, amides, and more polar compounds in general. (TIF) [file pone.0283259.s004.tif]

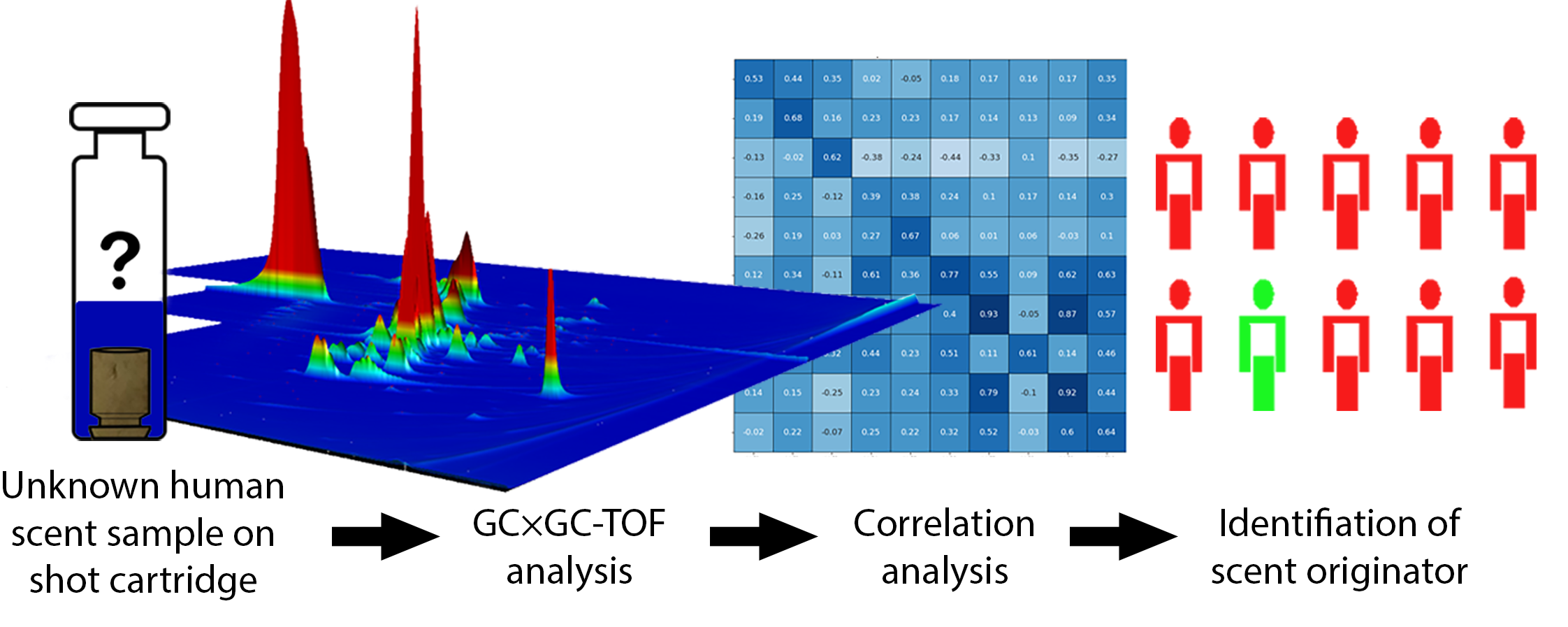

Supplement: S1 Graphical abstract — (TIF) [file pone.0283259.s008.tif]
