# Supplementary material for: Human scent signature on cartridge case survives gun being fired: A preliminary study on a potential of scent residues as an identification tool
Source: PLoS One. 2023 Mar 22;18(3):e0283259. doi: 10.1371/journal.pone.0283259 (PMC10032514; doi:10.1371/journal.pone.0283259)
Supplement: Attachment — Submitted filename: Response to Reviewers.docx [file pone.0283259.s009.docx]

**Response to Reviewers:**

Manuscript: Human Scent Signature on Cartridge Case Survives Gun Being Fired: A Preliminary Study on a Potential of Scent residues as an Identification Tool (PLOS ONE)

- p. 6, line 120, “all n detected peaks”. What is the value of n?

The argument 'all *n detected peaks'* was rewritten to 'all *75 selected peaks'.*

- p. 6, line 129, “the N number of peak area ratios with the least standard deviation (sorted in ascending order) through all samples were included in further calculations”. 1) Is it an intrasubject standard deviation? 2) How is N determined?

1) Yes, is the intrasubject SD
2) N is a parameter chosen by the scientist responsible for the experiment. The value can be set as an integer (rigid count of SDs from each subset will be chosen), or SD value threshold can be set (then the number of the SDs will most likely vary among the subsets). We have chosen the first approach, because we found the second approach more viable for normally distributed dataset. The comment was added to the body of the article [lines 157- 160]

- p. 6, line 131, “the incidence rate threshold was established”. 1) How ? 2) why is a second incidence rate threshold needed (the first one being 75% for each subject)?

1) This parameter is also chosen by the scientist responsible for the experiment. The lower tolerance to zero values means that only the common chemical compounds are kept for further analysis. The higher tolerance to zero values, on the other hand, involves even the subject-specific chemical compounds in the following analysis [lines 148 – 150].
2) The second incidence rate threshold was established because we wanted to eliminate subject-specific chemical compounds and keep only chemical compounds common for all (or majority of) subjects.

- • p. 6, line 134, “in the presented PCA models”. What PCA models? Figures 3 and 5 display score plots on the two first principal components, and the captions the percentage of the variance explained by the first 7 principal components. What is relevant to the selection?

Only the first two components were demonstrated, because they describe most of the variance. The rest of the percentage of described variance is just for informational reasons. Recalculation of the PCA models was done with a code provided to make it accessible for every reader. Another file (Calculations) was added to Supporting Information and described the models in more depth with the code provided.

- • p. 7, line 138: why is normality tested for?

It is another piece of information that we used to support our results. The finding that our data are not normal corresponds to a higher success rate of Spearman’s and Kindal’s correlations. It was very helpful when deciding how to choose N (mentioned in the previous question)

- • p. 7, line 140: “PCA [14] was applied as a model-based method”. If the authors mean that they evaluate the performance as the percentage of variance explained by the m first components, this should be stated (with the value of m). The selection of N and of the incidence rate threshold should be clarified here.

The statements were clarified in the body of the text (lines 162–166)

- p. 9, line 198: Figure 3 instead of 2.

Fixed numbering.

- p. 11, line 230: Figure 5 instead of 4.

Fixed numbering.

- p. 11, Fig. 5 caption: “Model with 50 ratios with least SD and with equal or less that 75% zero value for each ratio through the standard and fired samples (57 ratios in total) using leave-one out validation”: 1) how can 50 selected ratios become 57? 2) Since this model is based on the standard samples, why is the occurrence rate through standard AND fired samples?

1) 50 ratios from each volunteer (5 volunteers in total). The final set contained 57 unique ratios. Theoretically, if all volunteers had unique ratios that were not shared with the rest, we would end up with 250 unique ratios in our model.
2) We took every possible step to ensure that the relevant ratios for the final similarity comparison are present in both the standard and the scent samples.

- p. 11, section “Similarity analysis of fired cartridge samples versus standard scent samples”: which one of the two models is used, that based on the scent residue samples, or that based on the standard samples? The text does not tell, nor does the diagram of Figure 2.

Both ‘Similarity analyses’ were transformed into subsections of the corresponding model sections to improve readability.

- p. 13, line 276: “The model 75_50 () performed with the same results for Pearson’s correlation, cosine similarity and Kendall’s tau.” What results? How to they compare to Spearman’s? Especially the cosine similarity whose performance is very close to Spearman’s according to Table E.1.

Yes, if we look at the average values, the CS is doing as well as SC. However, if we look closely at Fig 6, the absolute difference between the average correlation value of class 1 (samples from the same volunteer) and class 0 (samples from different volunteers) was greater for Spearman’s than for cosine.

- p. 13, line 278, “The Spearman’s correlation reached a 100% success rate”: it is true, when looking what standard scent sample best matches a given fired cartridge sample, but not conversely (vol. 4 is matched with vol. 6).

The reviewer is right; we added this statement into the corresponding section and Conclusion.
