# Supplementary material for: Human scent signature on cartridge case survives gun being fired: A preliminary study on a potential of scent residues as an identification tool
Source: PLoS One. 2023 Mar 22;18(3):e0283259. doi: 10.1371/journal.pone.0283259 (PMC10032514; doi:10.1371/journal.pone.0283259)
Supplement: Attachment — Submitted filename: Response to Reviewers.docx [file pone.0283259.s010.docx]

**1^ST^ REVISION**

**-------------------------------------------------------------------------------------------------**

**Response to Reviewers:**

Manuscript: Human Scent Signature on Cartridge Case Survives Gun Being Fired: A Preliminary Study on a Potential of Scent residues as an Identification Tool (PLOS ONE)

- p. 6, line 120, “all n detected peaks”. What is the value of n?

The argument 'all *n detected peaks'* was rewritten to 'all *75 selected peaks'.*

- p. 6, line 129, “the N number of peak area ratios with the least standard deviation (sorted in ascending order) through all samples were included in further calculations”. 1) Is it an intrasubject standard deviation? 2) How is N determined?

1) Yes, is the intrasubject SD
2) N is a parameter chosen by the scientist responsible for the experiment. The value can be set as an integer (rigid count of SDs from each subset will be chosen), or SD value threshold can be set (then the number of the SDs will most likely vary among the subsets). We have chosen the first approach, because we found the second approach more viable for normally distributed dataset. The comment was added to the body of the article [lines 157- 160]

- p. 6, line 131, “the incidence rate threshold was established”. 1) How ? 2) why is a second incidence rate threshold needed (the first one being 75% for each subject)?

1) This parameter is also chosen by the scientist responsible for the experiment. The lower tolerance to zero values means that only the common chemical compounds are kept for further analysis. The higher tolerance to zero values, on the other hand, involves even the subject-specific chemical compounds in the following analysis [lines 148 – 150].
2) The second incidence rate threshold was established because we wanted to eliminate subject-specific chemical compounds and keep only chemical compounds common for all (or majority of) subjects.

- • p. 6, line 134, “in the presented PCA models”. What PCA models? Figures 3 and 5 display score plots on the two first principal components, and the captions the percentage of the variance explained by the first 7 principal components. What is relevant to the selection?

Only the first two components were demonstrated, because they describe most of the variance. The rest of the percentage of described variance is just for informational reasons. Recalculation of the PCA models was done with a code provided to make it accessible for every reader. Another file (Calculations) was added to Supporting Information and described the models in more depth with the code provided.

- • p. 7, line 138: why is normality tested for?

It is another piece of information that we used to support our results. The finding that our data are not normal corresponds to a higher success rate of Spearman’s and Kindal’s correlations. It was very helpful when deciding how to choose N (mentioned in the previous question)

- • p. 7, line 140: “PCA [14] was applied as a model-based method”. If the authors mean that they evaluate the performance as the percentage of variance explained by the m first components, this should be stated (with the value of m). The selection of N and of the incidence rate threshold should be clarified here.

The statements were clarified in the body of the text (lines 162–166)

- p. 9, line 198: Figure 3 instead of 2.

Fixed numbering.

- p. 11, line 230: Figure 5 instead of 4.

Fixed numbering.

- p. 11, Fig. 5 caption: “Model with 50 ratios with least SD and with equal or less that 75% zero value for each ratio through the standard and fired samples (57 ratios in total) using leave-one out validation”: 1) how can 50 selected ratios become 57? 2) Since this model is based on the standard samples, why is the occurrence rate through standard AND fired samples?

1) 50 ratios from each volunteer (5 volunteers in total). The final set contained 57 unique ratios. Theoretically, if all volunteers had unique ratios that were not shared with the rest, we would end up with 250 unique ratios in our model.
2) We took every possible step to ensure that the relevant ratios for the final similarity comparison are present in both the standard and the scent samples.

- p. 11, section “Similarity analysis of fired cartridge samples versus standard scent samples”: which one of the two models is used, that based on the scent residue samples, or that based on the standard samples? The text does not tell, nor does the diagram of Figure 2.

Both ‘Similarity analyses’ were transformed into subsections of the corresponding model sections to improve readability.

- p. 13, line 276: “The model 75_50 () performed with the same results for Pearson’s correlation, cosine similarity and Kendall’s tau.” What results? How to they compare to Spearman’s? Especially the cosine similarity whose performance is very close to Spearman’s according to Table E.1.

Yes, if we look at the average values, the CS is doing as well as SC. However, if we look closely at Fig 6, the absolute difference between the average correlation value of class 1 (samples from the same volunteer) and class 0 (samples from different volunteers) was greater for Spearman’s than for cosine.

- p. 13, line 278, “The Spearman’s correlation reached a 100% success rate”: it is true, when looking what standard scent sample best matches a given fired cartridge sample, but not conversely (vol. 4 is matched with vol. 6).

The reviewer is right; we added this statement into the corresponding section and Conclusion.

**2^ND^ REVISION**

**-------------------------------------------------------------------------------------------------**

**Reviewer's Responses to Questions:**

4. Have the authors made all data underlying the findings in their manuscript fully available?

Reviewer #1: No

We added the summary data (which were the starting point of our calculations) to a submission. All scripts used in the manuscript were already published in an open repository via Github.

5. Is the manuscript presented in an intelligible fashion and written in standard English?

Reviewer #1: No

The typos were checked via software, and we consulted the text with a native speaker. Can the Reviewer elaborate a bit on this issue?

6. Review Comments to the Author:

There is still no section numbering.

We even checked already published articles. They have no section numbering. The guidelines, and the sample manuscript body do not mention section numbering, sample manuscript has no numbering.

The notations for the parameters and models are still unclear.

We changed the notations of incidence thresholds to fix the problem with the notations. We have rewritten the incidence threshold to be corresponding with the used script as it was misleading. In the script, the user input is set to:

zerocount = int(input("Mow many zeros in column are acceptable? [%] "))

The incidence threshold should be referring to zero values. This issue is addressed in the body of the text.

Models are already referred in the text with the section reference as well as with the code. We added the naming policy to the body of the text [lines 152-153], hopefully it will help with the navigation. Both similarity comparison sections are already a subsection of the Model sections, so it is clear which model is used for the data pre-processing (because the PCA is not an identification tool, just a visualization).

• p. 6, line 120, “all n detected peaks”. What is the value of n?
Answer in the response to the reviewers : “The argument 'all n detected peaks' was rewritten to 'all 75 selected peaks'.”
Answer in the revised manuscript : n vanished, we read on p. 6 lines 132-134: “After data alignment via RIs, all detected peaks were inspected, and the subset of 75 selected chemical compounds presented in standard and fired sample datasets was created and further processed.” 'all 75 selected peaks' appears nowhere.

Reviewer is right, the original subset involved 101 peaks. The author was referring to a subset of 75 peaks in the Responses (after we excluded peaks appearing only in few samples). The Author is sorry for the confusion. ‘75 peaks’ is now in the text. The original summary table is split into three sections according to the samples and attached to the manuscript to improve point 5. Please note that the table containing only cartridge samples contains 59 peaks as 16 peaks had zero values in all cartridge samples.

So is the percentage of variance explained by the with 7 PC of a PCA model used to select the value of N? If yes, why 7? What configurations of N and of the two incidence rate ratios were tested?

No, the percentage of variance was not as significant as the distance and separation of each observed cluster on the final plot. We clearly stated that PCA was solely explorative tool. The higher the total explained variance, the better of course. However, the distance of the Volunteers clusters was the crucial parameter. First incidence rate was always set to 75% [line 141], the second one was set in range from 25 to 75 with step of 25. The N parameter was tested in range from 25 up to 500 with step of 25. We added the ranges into the body of the text.

Furthermore, some corrections/additions in the revised manuscript raise more questions than they provide clarifications. For example:

• p. 7, lines 147-148: “This threshold was also estimated and optimized.” This isolated sentence is added without any reference to the section where the optimization takes place.

This refers to the second incidence rate threshold.

Etc.

This is not a proper revision.

We decided to do an additional statistical analysis via KNN and RN to support our concept, because we feel from the Reviewer’s comments that explorative PCA as proof of the concept is not enough. For the final comparison, we added the test for significant differences for the top two correlation coefficients in the final sample comparison.

We hope this will improve the soundness of the results from the statistical point of view.
